# Inhibition of the Renin–Angiotensin System Improves Hemodynamic Function of the Diabetic Rat Heart by Restoring Intracellular Calcium Regulation

**DOI:** 10.3390/biomedicines13030757

**Published:** 2025-03-20

**Authors:** Krisztina Anna Paulik, Tamás Ivanics, Gábor A. Dunay, Ágnes Fülöp, Margit Kerék, Klára Takács, Zoltán Benyó, Zsuzsanna Miklós

**Affiliations:** 1 Institute of Translational Medicine, Semmelweis University, 1094 Budapest, Hungary; paulik.krisztina@phd.semmelweis.hu (K.A.P.); ivanics.tamas@semmelweis.hu (T.I.); gadunay@protonmail.com (G.A.D.); fulop.agnes@semmelweis.hu (Á.F.); claritakacs@gmail.com (K.T.); benyo.zoltan@semmelweis.hu (Z.B.); 2 Klinikum Westbrandenburg, Brandenburg Medical School (MHB), 14770 Brandenburg an der Havel, Germany; 3 Faculty of Health Sciences, Joint Faculty of the Brandenburg University of Technology Cottbus-Senfteberg, the Brandenburg Medical School Theodor Fontane and the University of Potsdam, 14476 Potsdam, Germany; 4 HUN-REN-SU Cerebrovascular and Neurocognitive Diseases Research Group, 1094 Budapest, Hungary; 5 National Korányi Institute for Pulmonology, 1122 Budapest, Hungary

**Keywords:** renin–angiotensin system (RAS), losartan, enalapril, Langendorff heart, diabetic cardiomyopathy, type 1 diabetes, type 2 diabetes, cardiac Ca^2+^_i_ transient, Indo-1 surface fluorometry, SERCA2a

## Abstract

**Background/Objectives:** Disrupted intracellular calcium (Ca^2+^_i_) regulation and renin–angiotensin system (RAS) activation are pathogenetic factors in diabetic cardiomyopathy, a major complication of type 1 (T1D) and type 2 (T2D) diabetes. This study explored their potential link in diabetic rat hearts. **Methods:** Experiments were conducted on T1D and T2D Sprague-Dawley rats induced by streptozotocin and fructose-rich diet, respectively. In T1D, rats were treated with Enalapril (Ena) or Losartan (Los) for six weeks, whereas T2D animals received high-dose (HD) or low-dose (LD) Ena for 8 weeks. Heart function was assessed via echocardiography, Ca^2+^_i_ transients by Indo-1 fluorometry in Langendorff-perfused hearts, and key Ca^2+^_i_ cycling proteins by Western blot. Data: mean ± SD. **Results:** Diabetic hearts exhibited reduced contractile performance that was improved by RAS inhibition both in vivo (ejection fraction (%): T1D model: Control: 79 ± 7, T1D: 54 ± 11, T1D + Ena: 65 ± 10, T1D + Los: 69 ± 10, *n* = 18, 18, 15, 10; T2D model: Control: 73 ± 8, T2D: 52 ± 6, T2D + LDEna: 62 ± 8, T2D + HDEna: 76 ± 8, *n* = 9, 8, 6, 7) and ex vivo (+dPressure/dt_max_ (mmHg/s): T1D model: Control: 2532 ± 341, T1D: 2192 ± 208, T1D + Ena: 2523 ± 485, T1D + Los: 2643 ± 455; T2D model: Control: 2514 ± 197, T2D: 1930 ± 291, T2D + LDEna: 2311 ± 289, T2D + HDEna: 2614 ± 268). Analysis of Ca^2+^_i_ transients showed impaired Ca^2+^_i_ release and removal dynamics and increased diastolic Ca^2+^_i_ levels in both models that were restored by Ena and Los treatments. We observed a decrease in sarcoendoplasmic reticulum Ca^2+^-ATPase2a (SERCA2a) expression, accompanied by a compensatory increase in 16Ser-phosphorylated phospholamban (P-PLB) in T2D that was prevented by both LD and HD Ena (expression level (% of Control): SERCA2a: T2D: 36 ± 32, T2D + LDEna: 112 ± 32, T2D + HDEna: 106 ± 30; P-PLB: T2D: 557 ± 156, T2D + LDEna: 129 ± 38, T2D + HDEna: 108 ± 42; *n* = 4, 4, 4). **Conclusions:** The study highlights the critical role of RAS activation, most likely occurring at the tissue level, in disrupting Ca^2+^_i_ homeostasis in diabetic cardiomyopathy. RAS inhibition with Ena or Los mitigates these disturbances independent of blood pressure effects, underlining their importance in managing diabetic heart failure.

## 1. Introduction

Diabetes mellitus is a common disease, and its prevalence in Western societies has an increasing trend [1,2]. A strong association between diabetes mellitus and heart failure has been described [3,4,5].

Several studies have reported that the incidence of heart failure is higher in diabetic patients [5,6,7,8,9]. This is primarily linked to the development of diabetic cardiomyopathy, a condition recognized for over 50 years and quite common in both type 1 (T1D) and type 2 diabetes (T2D) [10]. The typical alterations in myocardial structure and function characterizing diabetic cardiomyopathy are not necessarily associated with other comorbidities, such as hypertension or coronary artery disease, and typically manifest in the form of an early diastolic dysfunction and left ventricular hypertrophy, followed by cardiac fibrosis and a progressive loss of systolic performance [11]. Hyperglycemia and insulin resistance are both thought to play important roles in driving diabetes-related cardiac pathophysiology that involves shifts in substrate metabolism, lipid toxicity, oxidative stress, maladaptive neurohormonal regulation, altered intracellular calcium (Ca^2+^_i_) cycling, and protein expression profile [11,12,13]. Despite its clinical significance, diabetic cardiomyopathy has no currently existing specific therapy. Therefore, further research is needed to better understand the role of available heart failure therapies in the management of this disease entity [12].

The role of the activation of the renin–angiotensin system (RAS) in the pathogenesis of heart failure is widely recognized [14,15,16,17,18]. The details of the classical pathway of systemic RAS activation have been known for decades, alongside which local RAS pathways have been described in various tissues. These pathways function independently of each other and the systemic RAS, playing fundamental roles in the pathophysiology of various diseases [19,20]. In the heart, angiotensin II (ATII), through its action on angiotensin type 1 receptors (AT1Rs), induces cellular hypertrophy, fibroblast activation, and collagen deposition, which contribute to myocardial fibrosis and remodeling—key features in the progression of heart failure [19]. Chronic AT1R activation by ATII ignites complex maladaptive cell growth and hypertrophic responses that involve phospholipase C (PLC) mediated activation of various downstream pathways, such as calcineurin and mitogen-activated protein kinase (MAPKs) pathways that regulate transcription factors like nuclear factor-kappa B (NF-κB) and STAT proteins. In addition, AT1R activation induces oxidative stress by triggering NADPH oxidase (NOX) activity and mitochondrial dysfunction [19,21]. Insulin deficiency and hyperglycemia have been shown to contribute to local cardiac RAS activation in various animal models, also highlighting its potential pathological role in the development of diabetic cardiomyopathy-associated heart failure [14,15,16,17,18,19]. Furthermore, evidence from animal studies is supported by observations from human studies, which reported beneficial effects of AT1R blockers and angiotensin-converting enzyme (ACE) inhibitors in treating diabetic patients with heart failure [22,23]. However, important details about the connection between RAS activation and the progression of diabetic heart failure remain unclear.

One of the characteristics of diabetic cardiomyopathy is altered calcium signaling [24] observed in various experimental models, including the isolated perfused heart preparation [25]. Disturbances of Ca^2+^_i_ levels in cardiomyocytes are fundamental points of failure of the contraction/relaxation cycle in cardiomyopathy [26]. Insulin deficiency and hyperglycemia are indicated to be crucial components of increasing the stress of the endoplasmic reticulum, a key organelle in Ca^2+^_i_ cycling [12]. In diabetes, reduced expression of transporters and channels responsible for Ca^2+^_i_ homeostasis (sarcoendoplasmic reticulum Ca^2+^-ATPase 2a (SERCA2a), the ryanodine receptor type 2 (RyR2), the sarcolemma Na^+^-Ca^2+^ exchanger, and the L-type Ca^2+^ channel) may be due to the lack of insulin or insulin effect as a growth factor [24,27,28,29]. Not only the expression but also the function of these Ca^2+^_i_ handling proteins is impaired, leading to inadequate Ca^2+^ uptake and Ca^2+^ sequestration [7,8,9,12,26,29,30]. Inadequate functions of the SERCA2a transporter of cardiomyocytes in an animal model of metabolic syndrome have been published by our research group [29,31]. Ca^2+^_i_ levels were also found to be elevated in isolated diabetic cardiomyocytes. Since RAS activation leads to an increased oxidative stress response and oxidative stress has been shown to disturb Ca^2+^_i_ homeostasis in cardiomyocytes and lead to elevated Ca^2+^_i_ levels [32,33], it is feasible to postulate a relationship between RAS activation and impaired Ca^2+^_i_ handling in the diabetic heart.

Although experimental data highlight that activation of RAS and disruption of the Ca^2+^_i_ homeostasis in cardiac tissue both play an important role in the development of diabetic cardiomyopathy, evidence supporting a close relationship between them is scarce in the literature. Furthermore, it is also unclear whether RAS inhibitor treatment, commonly used in heart failure therapy, has protective effects on Ca^2+^_i_ homeostasis of the diabetic heart.

In the present study, our main objective was to explore the potential link between RAS activation and impaired Ca^2+^_i_ handling in experimental rat models of diabetes. To achieve this, we administered chronic RAS inhibitor treatment using the ACE-blocker Enalapril (Ena) and AT1R blocker Losartan (Los) to diabetic rats. We then evaluated their effects on heart function both in vivo and ex vivo, utilizing echocardiography and the Langendorff perfused heart preparation, respectively. In addition, we recorded Ca^2+^_i_ transients in the isolated beating heart preparation by Indo-1 surface fluorometry and analyzed the effects of chronic RAS inhibitor treatment on Ca^2+^_i_ cycling dynamics of the diabetic heart.

Using these main methodological approaches, first, we explored if chronic treatment with Ena or Los can prevent the diabetes-related disruption of Ca^2+^_i_ handling in a streptozotocin (STZ) induced type 1 diabetic model. Secondly, we used a fructose-diet-induced T2D model to investigate whether ACE inhibitor treatment by Ena, commonly used to treat hypertension in T2D, provides benefits for cardiac Ca^2+^_i_ handling in diabetic heart failure, and if these benefits occur independently of blood pressure reduction, potentially indicating the relevance of local RAS in the process. To investigate this, we administered chronic Ena treatment at both high (depressor) and low (subdepressor) doses in a fructose-fed rat model.

## 2. Materials and Methods

### 2.1. Study Design

#### 2.1.1. Animal Models and Ethical Considerations

**Figure 1:**
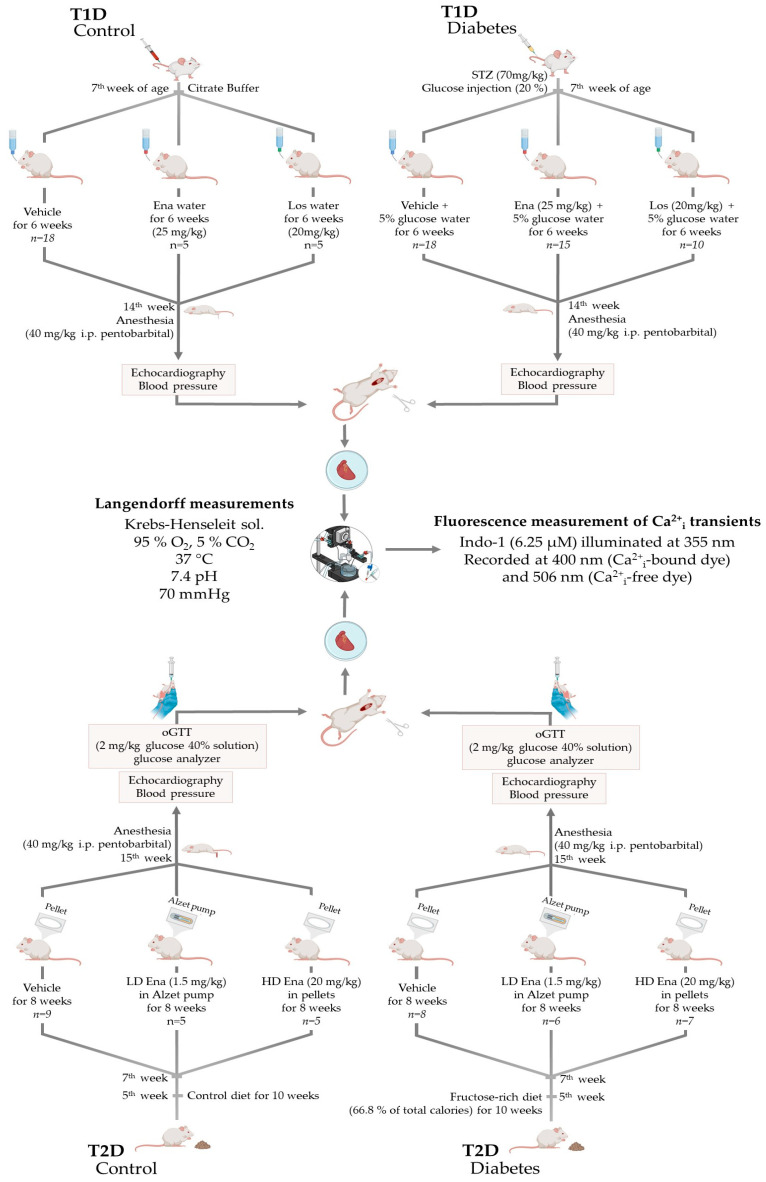
The T1D and T2D animal models and the experimental protocol. Abbreviations: T1D—type 1 diabetes; T2D—type 2 diabetes; STZ—streptozotocin injection; Ena—Enalapril; Los—Losartan; LD Ena—low-dose Enalapril; HD Ena—high-dose Enalapril; oGTT—oral glucose tolerance test.

Experiments were carried out on T1D and T2D male adult Sprague-Dawley rats. The rats were housed in a 12 h light and 12 h dark cycle and had free access to food and water in the animal facility of Basic Medical Science Center, Semmelweis University, Budapest, Hungary. All applied procedures conform to the guidelines of the Hungarian Law of Animal Protection (28/1998) and Decree No. 40 of 2013 (II. 14.) of the Hungarian Government on animal testing and were approved and authorized by the Government Office of Pest County (Permission number: PEI/001/820-2/2015 date: 26 February 2015; and PEI/001/803-2/2015 date: 2 March 2015). In compliance with guidelines, the animals were handled humanely, with all measures taken to minimize their suffering and the number of animals used in experiments. Model- and procedure-specific measures are detailed later in the text, where the animal models are discussed. Details of the models and the experimental groups of T1D and T2D are displayed in Figure 1 and described below.

#### 2.1.2. Type 1 Diabetic Model (T1D)—Experimental Design

Male Sprague-Dawley rats (*n* = 75) at the age of 8 weeks weighing 200–250 g were enrolled in the T1D study (Figure 1. Top panel). They were randomly assigned to T1D (*n* = 45) and T1D Control (*n* = 30) experimental arms. Before T1D induction, animals were anesthetized with an injection mixture of ketamine (Calypsol 50 mg/mL inj., Richter Gedeon Plc., Budapest, Hungary) and xylazine (Merck Group, Darmstadt, Germany) (37.5 mg/kg and 7.5 mg/kg, respectively) to minimize the traumatic stress of the intervention. In the T1D arm, T1D was induced by a single dose of 70 mg/kg streptozotocin (STZ) (Merck Group, Darmstadt, Germany) administered by intravenous injection into the tail vein. STZ injection was prepared right before administration by dissolving 30 mg/mL STZ in disodium citrate buffer (0.01 mM, pH 4.5 Buffer Solution pH 4.5, Sigma-Aldrich Cas. No.: 82565, Merck Group, Darmstadt, Germany—product is now obsolete). STZ was used to destroy pancreatic ß-cells. After injection, rats were also given a single injection of 5 mL of 20% glucose subcutaneously to prevent hypoglycemic coma. Rats of the T1D Control arm underwent the same procedure but received only vehicle [25,31].

After T1D induction, the animals were housed with 2 per cage. Special care was given to T1D rats, and their litter was changed daily after the onset of polyuria to minimize discomfort and suffering. T1D rats were provided with a 5% glucose solution to drink starting from the day of the STZ treatment. In both experimental arms, the animals were divided into three subgroups. The T1D arm was as follows:

- Fifteen animals received ACE inhibitor Ena (Enalapril maleate salt, Sigma-Aldrich Cas. No.: E-6888; Merck Group, Darmstadt, Germany) at a therapeutic dosage of ~25 mg/kg [34,35] in their drinking solution (T1D + Ena group, *n* = 15);
- The drinking solution of 10 animals was supplemented with AT1R antagonist Los (Losartan Potassium, generous gift from Richter Gedeon Plc., Budapest, Hungary) at a dosage of ~20 mg/kg [36] (T1D + Los group, *n* = 10).
- Twenty rats consumed the glucose solution without drug supplementation (T1D Control, *n* = 20). However, two animals did not survive due to acute T1D complications, leaving 18 for analysis (T1D Control group, *n* = 18).

The Control arm was as follows:

- Twenty animals served as the negative Control group; however, in 2 cases, the isolated heart preparation was not successful, reducing the final sample size to eighteen (Control, *n* = 18).
- To assess the potential effects of Ena and Los in healthy animals, 2 additional subgroups were created with 5 animals in each, where rats received the respective treatments (Control + Ena, *n* = 5 and Control + Los, *n* = 5).

The treatment began the day after the induction of T1D and lasted 6 weeks. After 6 weeks, all animals underwent the experimental protocol detailed in Section 2.2.

#### 2.1.3. Type 2 Diabetic Model (T2D)—Experimental Design

Male Sprague-Dawley rats (*n* = 43) at the age of 5 weeks were randomly enrolled in 2 experimental arms: T2D (*n* = 24) and T2D Control (*n* = 20) (Figure 1, lower panel). In the T2D arm, rats were fed a fructose-rich diet for 10 weeks to induce insulin resistance. The diet consisted of 66.8% of total calories from fructose (custom research diet No. TD.06451 by Harlan Teklad, Madison, WI, USA). In the T2D Control arm, rats of the same age consumed the Control diet recommended by the manufacturer, in which carbohydrate, protein, and fat contents were adjusted to the values of the research diet (diet No. TD.06452 by Harlan Teklad, Madison, WI, USA) [29].

Two weeks after the initiation of the research diet (at 7th week of age), animals underwent surgery to implant subcutaneous drug releasers. We created 3 subgroups within both experimental arms. The T2D arm was as follows:

- One of the subgroups constituted animals (*n* = 8) treated with high-dose (HD) ACE inhibitor Ena (T2D + HD Ena group). This substance was liberated for 8 weeks continuously from subcutaneous biodegradation pellets. For this purpose, two 60-day release pellets of 200 mg Ena total dose (Innovative Research, Novi, MI, USA; Catalogue No.: SC-999) were implanted below the skin of the back to ensure an approximately 20 mg/kg mean daily dosage of the substance. Due to technical issues during the Langendorff experiment, one individual was excluded from the study, reducing the final sample size to 7.
- Another subgroup (*n* = 8) received Ena in low-dose (LD) (1.5 mg/kg [37,38], T2D + LD Ena group) via osmotic minipumps inserted below the skin of the back for 8 weeks. For this purpose, 4-week release 2 mL Alzet pumps (“2ML4” Alzet osmotic pump, DURECT Corporation, Cupertino, CA, USA) were filled with Ena (Enalapril maleate salt, Sigma-Aldrich Cas. No.: E-6888; Merck Group, Darmstadt, Germany) dissolved in distilled water to achieve the targeted mean daily release of 1.5 mg/kg. Pumps were replaced after 4 weeks to maintain a steady, even dose of the medicine. The LD Ena group was involved in the experimental protocol to inhibit local RAS activity by ACE inhibitor treatment independent of systemic blood pressure changes. The final sample size was 6, as 2 experiments were excluded due to cardiac arrest during isolated heart experiments.
- A subgroup of animals (*n* = 8) underwent the same operation procedure as the other subgroups, and subcutaneous pellets (*n* = 4) or osmotic minipumps (*n* = 4) loaded with vehicles were placed below their skins (T2D group). The experimental protocol was performed 10 weeks after the initiation of the fructose diet.

In the T2D Control arm, the same subgroups were created following the same procedures:

- T2D Control + HD Ena group (*n* = 5);
- T2D Control + LD Ena group (*n* = 5);
- T2D Control group (*n* = 9).

The surgical procedure was the same on each occasion. Rats were anesthetized with 40 mg/kg intraperitoneal pentobarbital injection (Euthasol 40%; Produlab Pharma BV, Raamsdonksveer, The Netherlands). After shaving and disinfection, a skin incision was made on the back, and biodegradation pellets/minipumps were inserted subcutaneously. The wound was then closed with surgical sutures. To prevent postoperative infections, animals received a single injection of amoxicillin + clavulanic acid (50 + 7.1 mg/kg; Augmentin—GSK, Budapest, Hungary). Following surgery, they were housed individually for two days and given ibuprofen (~30 mg/kg in drinking water; Merck Group, Darmstadt, Germany) for pain relief.

In each subgroup, the treatment was terminated 10 weeks after the initiation of the fructose diet, and the experimental protocol described in Section 2.2 was performed.

### 2.2. Experimental Protocol

At the end of the treatment protocols, animals of each experimental group were anesthetized with 40 mg/kg intraperitoneal pentobarbital injection (Euthasol 40%; Produlab Pharma BV, Raamsdonksveer, The Netherlands) and echocardiography was performed to evaluate heart dimensions and in vivo left ventricular functions (Figure 1). Echocardiography was followed by the measurement of arterial blood pressure with the help of a catheter inserted into the left carotid artery connected to a pressure transducer (Experimetria Ltd., Budapest, Hungary). To assess the diabetic status of the rats, blood glucose level was measured, and T2D animals were subjected to an oral glucose tolerance test (oGTT). In vivo measurements were followed by Langendorff preparation and perfusion of the isolated hearts along with concurrent fluorescent measurement of Ca^2+^_i_ transients in the heart tissue. Tissue samples of hearts from the T2D model experiments were quick-frozen to determine the expression of key Ca^2+^_i_ cycling enzymes with Western blot analysis.

#### 2.2.1. Echocardiography

Echocardiography was performed with a SONOS 5500 ultrasound machine (Hewlett Packard, Palo Alto, CA, USA) equipped with a high-frequency linear transducer (5–15 MHz). Long-axis B-mode images of the left ventricle were taken to calculate both end-systolic volume (ESV) and end-diastolic volume (EDV) from left ventricular area (LVA) and left ventricular length (LVL) as 8(LVAd)2/3πLVLd and 8(LVAs)2/3πLVLs, respectively (‘d’ and ‘s’ represent diastole and systole, respectively). From these data, the ejection fraction (EF) was determined as 100(EDV-ESV)/EDV. Short-axis echocardiograms were captured at the level of the papillary muscles to determine the diastolic wall thickness of the left ventricle and fractional shortening (FS). In order to assess FS, left ventricular internal diameters in diastole and systole were measured (LVIDd and LVIDs, respectively), and FS was calculated using the formula 100(LVIDd-LVIDs)/LVIDd [29,39].

#### 2.2.2. Blood Glucose Measurement, oGTT

Blood glucose levels of animals were measured from a venous blood sample using a Dcont Ideál blood glucose meter (Dcont Start, 77 Elektronika Ltd., Budapest, Hungary). In T1D experimental groups, random blood glucose level was determined before the Langendorff preparation.

In T2D experimental groups, animals were subjected to an oGTT after overnight fasting. For this purpose, 2 mg/kg glucose in 40% solution was administered via gastric gavage after determining fasting blood glucose level from a venous blood sample from a line placed into the jugular vein. In a subset of animals (*n* = 3–4 per group), serum samples were collected to determine fasting and 2 h insulin levels using an enzyme-linked immunosorbent assay (Invitrogen Rat Insulin ELISA Kit, Cat. No.: ERINS; Thermo Fisher Scientific, Waltham, MA, USA) (Figure 1).

#### 2.2.3. Isolated Langendorff Perfused Heart—Fluorescent Measurement of Ca^2+^_i_ Transients

Details of the concomitant measurements of hemodynamic parameters and fluorescence signals in a beating heart preparation were previously outlined [31,40]. In short, after echocardiography and blood pressure measurement, hearts were quickly removed from the body and mounted on a Langendorff perfusion apparatus and perfusion was initiated at constant-pressure mode (perfusion pressure: 70 mmHg) with a modified Krebs–Henseleit solution, containing (in mM) NaCl (118), KCl (4.3), NaHCO_3_ (25), KH_2_PO_4_ (1.2), MgSO_4_ (1.2), Na-EDTA (0.5), CaCl_2_ (2.0), pyruvate (5) and glucose (11) (all chemicals purchased from Merck Group, Darmstadt, Germany), and equilibrated with 5% CO_2_ and 95% O_2_. The pH of the buffer was adjusted to 7.4, and its temperature was maintained at 37 °C. Left ventricular pressure (LVP) changes were recorded with a balloon catheter, which was placed into the left ventricle and connected to a pressure transducer (Experimetria Ltd., Budapest, Hungary). Diastolic pressure was set at 8 mmHg to maintain constant preload. Coronary flow was monitored by an ultrasonic transducer (custom-calibrated Transonic 2PXN flow probe, Transonic Systems Inc., Ithaca, NY, USA) inserted in the perfusion line. Devices were connected to a computer, and data were recorded and analyzed using Haemosys Advanced 3.2 software (Experimetria Ltd., Budapest, Hungary). From continuous LVP curves, heart rate (HR) was calculated, and the positive and negative maximum values of the first derivative of the LVP (+dLVP/dt_max_, −dLVP/dt_max_) were determined as indices of left ventricular inotropic and lusitropic performance, respectively [41,42].

Details of the detection of Ca^2+^_i_ transients using Indo-1 surface fluorometry have already been published elsewhere [31,40]. Briefly, after recording baseline tissue autofluorescence, hearts were infused with the Ca^2+^ sensitive fluorescent dye by perfusing the coronaries with modified Krebs–Henseleit solution containing 6.25 μM Indo-1 AM (Invitrogen—Thermo Fisher Scientific, Waltham, MA, USA) in recirculating mode with a peristaltic pump (Cole-Palmer, Chicago, IL, USA) connected to the aortic cannula. Dye loading lasted for 20 min and was performed at a flow rate of 70% of baseline coronary flow. The temperature was maintained at 30 °C during the loading process. After dye loading, normal Langendorff perfusion was reestablished with the modified Krebs–Henseleit buffer. This solution was supplemented with 0.6 mM probenecid (Merck Group, Darmstadt, Germany) to inhibit dye-leakage from the cardiomyocytes. Indo-1 loaded hearts were allowed to stabilize for 20 min, after which fluorescence signals were recorded together with coronary flow and left ventricular pressure parameters.

Dye-loaded hearts were illuminated at 355 nm by a mercury arc lamp. The light emitted by Indo-1 was recorded at 400 nm (Ca^2+^-bound dye) and 506 nm (Ca^2+^-free dye) using a custom-made fluorometer. The fluorescence signals were recorded and stored for off-line analysis (Haemosys Advanced 3.2 software, Experimetria Ltd., Budapest, Hungary). Ca^2+^_i_-transient data were calculated using the ratiometric technique from 400 nm and 506 nm signals. The individual fluorescence signals were corrected for closed shutter background and tissue autofluorescence of the unloaded tissue. We used three unloaded hearts to test the changes in autofluorescence signals, which did not alter significantly from baseline values at either wavelength throughout the experimental procedure. Ca^2+^_i_ concentration was determined using the formula determined by Grynkiewicz et al., as described previously [31,43].

The Ca^2+^_i_ transient was assessed to determine systolic and diastolic Ca^2+^_i_ and Ca^2+^_i_ amplitude. The maximal rate of rise (+dCa^2+^_i_/dt_max_) and decline of Ca^2+^_i_ (−dCa^2+^_i_/dt_max_) were obtained as indices for Ca^2+^ release and sequestration from and back to the SR, respectively.

#### 2.2.4. Determination of Key Ca^2+^_i_ Cycling Enzymes in T2D Groups by Western Blot Analysis

In the experimental groups of the T2D model, protein expression of key regulators in Ca^2+^_i_ release and sequestration, namely that of the cardiac isoform of ryanodine channel (RYR2), SERCA2a, its main regulator phospholamban (PLB), and the 16Ser-phosphorylated form of PLB (P-PLB) was determined by conventional Western blot technique [29]. For this purpose, cardiac left ventricles were quick-frozen in liquid nitrogen and stored at −80 °C until analysis. Left ventricular tissue was sliced with a razor, and 1 g tissue was mixed with 3 mL radioimmunoprecipitation assay (RIPA) Buffer (R0278, Merck Group, Darmstadt, Germany). Samples were then homogenized with a manual homogenizer on ice until the tissue was liquefied. After centrifugation at 13,000 rpm, the supernatant was collected. Protein concentrations were determined using the Bradford Protein Assay. Tissue lysates were mixed in 2X Laemmli sample buffer and boiled for 5 min at 95 °C. Rather than being boiled, samples for P-PLB determination were kept at room temperature following the instructions of the manufacturer of the antibody. The samples were subjected to SDS-PAGE (7.5% gels were loaded with 20–25 µg protein per lane) and transferred to nitrocellulose membranes. Membranes were then blocked with 5% bovine serum albumin in tris-buffered saline containing 0.1% Tween 20 (TBST, Merck Group, Darmstadt, Germany) at room temperature for 1 h and incubated with the following primary antibodies: anti- Glyceraldehyde 3-phosphate dehydrogenase (GAPDH) (G-9) 1:1000; (sc-365062, Santa Cruz Biotechnology, Dallas, TX, USA), RyR2 1:1000; (Ab9080; Chemicon, Merck Group, Darmstadt, Germany), anti-SERCA2a Antibody 1:500; (NB100-237, Novus, St. Louis, MO, USA), anti-phospholamban 1:500; (FL-52, sc-30142, Santa Cruz Biotechnology, Dallas, TX, USA), anti-phospho-phospholamban 1:500 (Ser16) Antibody (07-052 Millipore, Burlington, MA, USA) overnight at 4 °C. After washing three times for 10 min with TBST, membranes were incubated with either Horseradish Peroxidase (HRP) conjugated anti-Mouse-IgG Polyclonal Goat IgG (HAF007, Biomedica, Wien, Austria) (for GADPH) or anti-rabbit IgG (sc-2004, Santa Cruz Biotechnology, Dallas, TX, USA) secondary antibodies in 1:5000 dilution, and signals were detected on Fuji Super RX films (Fujifilm, Budapest, Hungary) using the enhanced chemiluminescence (ECL) method (34077 SuperSignal West Pico Chemiluminescent Substrate, Thermo Fisher Scientific, Waltham, MA, USA). ImageJ software (version 1.53, National Institutes of Health, Bethesda, MD, USA) was used to compare the density of bands on Western blot. Optical density values obtained were normalized to those of the housekeeping enzyme, GAPDH, of the same sample. The normalized values of the Control hearts were set at 100%. Normalized densitometric values of independent experiments (*n* = 4 per group) were then expressed as a percentage of Control and averaged.

### 2.3. Statistical Analysis and Interpretation

The normality of the distribution of datasets was assessed using the Shapiro–Wilk test. For statistical comparison of experimental groups, one-way ANOVA (in case of normal distribution) and Tukey’s multiple comparison post hoc test or the equivalent Kruskal–Wallis non-parametric test (when normality test failed) complemented with Dunn’s multiple comparison tests were used. Every possible comparison between the study groups was considered. *p* < 0.05 was accepted throughout as a level of significance. The results are expressed as mean ± SD. Statistical analyses were performed using GraphPad Prism 8.0.1. (GraphPad, San Diego, CA, USA). During data analysis, no significant differences were observed between the data from the T1D Control + Los and T1D Control + Ena groups or between the T2D Control + LD Ena and T2D Control + HD Ena groups compared to their respective T1D Control and T2D Control groups. Therefore, for clarity in the graphs, only data from the untreated Control groups (T1D Control and T2D Control) are presented as Controls in both models.

## 3. Results

### 3.1. Biometric and Physiological Parameters in T1D and T2D Animals

Biometric measurements in the T1D model 6 weeks after T1D induction showed that body weight and proportional heart weight growth of rats were arrested following STZ treatment in the T1D group (Table 1, upper panel). Treatment with the ACE inhibitor Enalapril and the AT1R antagonist Losartan partially mitigated these effects; however, the growth rate did not fully return to its original levels. Mean arterial pressure did not alter in any of the experimental groups. Blood glucose levels confirmed severe hyperglycemia in the T1D group, which was not affected by treatments with Enalapril or Losartan (Table 1, upper panel).

In the T2D model, the body weights and fasting blood glucose levels were similar in all experimental groups (Table 1, lower panel). As typical of this animal model, high mean arterial pressure and increased heart weight and heart weight to body weight ratio (potentially indicating myocardial hypertrophy) were observed after 10 weeks on the fructose-rich diet. HD Ena, but not LD Ena, was able to prevent the development of hypertension and the increase in heart weight and heart weight to body weight ratio (Table 1, lower panel).

### 3.2. Blood Glucose and Insulin Levels in Response to oGTT in T2D Animals

**Figure 2:**
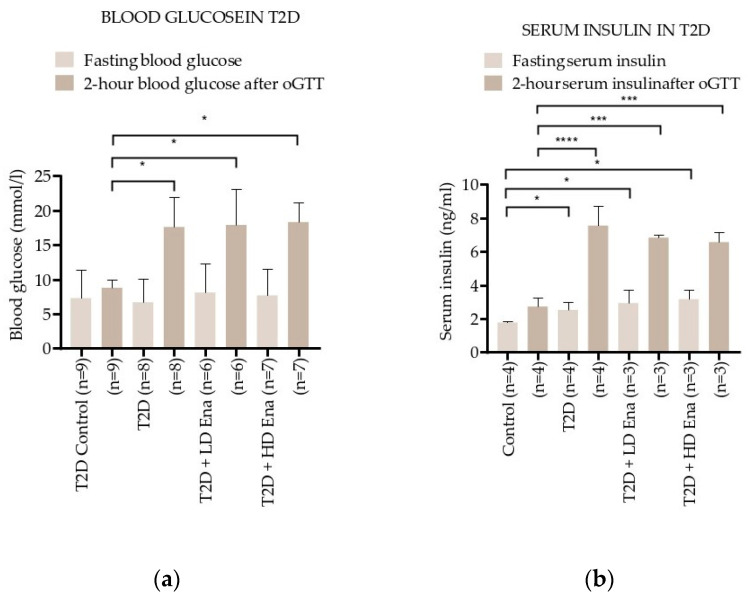
Changes in blood glucose and serum insulin levels during the oGTT test in T2D: Effect of T2D (*n* = 8), T2D + LD Ena (*n* = 6) and T2D + HD Ena (*n* = 7) on fasting blood glucose level and 2 h blood glucose level after oGTT (**a**); effect of T2D (*n* = 4), T2D + LD Ena (*n* = 3) and T2D + HD Ena (*n* = 3) on fasting serum insulin level and 2 h serum insulin level after oGTT (**b**). Statistics: Kruskal–Wallis ANOVA on ranks and Dunn’s multiple comparison test. * *p* < 0.05, *** *p* < 0.001 and ****: *p* < 0.0001 vs. T2D Control values (Mean ± SD). Abbreviations: T1D—type 1 diabetes, T2D—type 2 diabetes, Ena—Enalapril, Los—Losartan, LD Ena—low-dose Enalapril, HD Ena—high-dose Enalapril, oGTT—oral glucose tolerance test.

Figure 2a shows the effect of oGTT on blood glucose levels in the T2D model. Fasting blood glucose levels were in the normal range in all of the experimental groups, but the 2 h blood glucose levels at least doubled compared to Control values in T2D, T2D + LD Ena, and T2D + HD Ena groups (8.8 ± 1.2 vs. 17.6 ± 4.3, 17.9 ± 5.2 and 18.3 ± 2.9 mmol/L, respectively; *p* < 0.05) and showed no differences between untreated and treated diabetic animals.

Insulin levels were determined in a subgroup of animals (*n* = 3–4 per group), and their changes are illustrated in Figure 2b. Fasting serum insulin levels were higher in T2D compared to Control, and this increase was not prevented by either LD or HD Enalapril treatment (1.8 ± 0.1 vs. 2.5 ± 0.5, 3.2 ± 1.4 and 2.9 ± 1.2 ng/mL, respectively; *p* < 0.05). The serum insulin levels after 2 h oGTT almost tripled in group T2D compared to Control and were equally high in the T2D + LD Ena and the T2D + HD Ena groups (2.7 ± 0.5 vs. 7.6 ± 2.1, 6.6 ± 0.2 and 6.8 ± 0.1 ng/mL, respectively; *p* < 0.05). Data regarding serum insulin levels are consistent with blood glucose levels, indicating that the increased insulin resistance developing in response to high fructose diet was not corrected by either HD or LD Enalapril treatment (Figure 2b).

Although there was no statistical difference in the fasting blood glucose level between Control and T2D animals, the 2 h blood glucose level in oGTT in T2D groups remained considerably above 11.1 mmol/L, confirming the diabetic status of these rats [44,45].

### 3.3. Results of Echocardiography in T1D and T2D Animals

Table 2 summarizes the echocardiographic data both in T1D and T2D hearts.

Morphological parameters such as diastolic wall thickness and end-diastolic volume were not different from Controls in either of the T1D groups (Table 2, upper panel). However, the ejection fraction and fractional shortening values, which are indices of pump function, were significantly reduced in T1D. This reduction in contractile performance was significantly attenuated by both the ACE inhibitor Enalapril and the AT1R antagonist Losartan treatment (T1D + Ena and T1D + Los groups).

In the T2D model, diastolic wall thickness showed significantly higher values, which was not affected by LD Enalapril treatment but returned close to Control in response to HD Enalapril administration (Table 2, lower panel). These data are consistent with heart weight data (Table 1, lower panel). End-diastolic volume was not different from Controls in any of the T2D groups. The ejection fraction value decreased in T2D hearts, and this decrease was prevented completely by HD Enalapril treatment only (T2D + HD Ena group). With lower doses of Enalapril (T2D + LD Ena group), the ejection fraction also increased significantly but did not reach Control values. Fractional shortening displayed similar changes to ejection fraction, i.e., T2D significantly reduced fractional shortening, and this reduction was mitigated in response to LD Enalapril treatment and was completely prevented by HD Enalapril treatment (Table 2, lower panel).

### 3.4. Hemodynamic Parameters in Langendorff Hearts of T1D and T2D Animals

**Figure 3:**
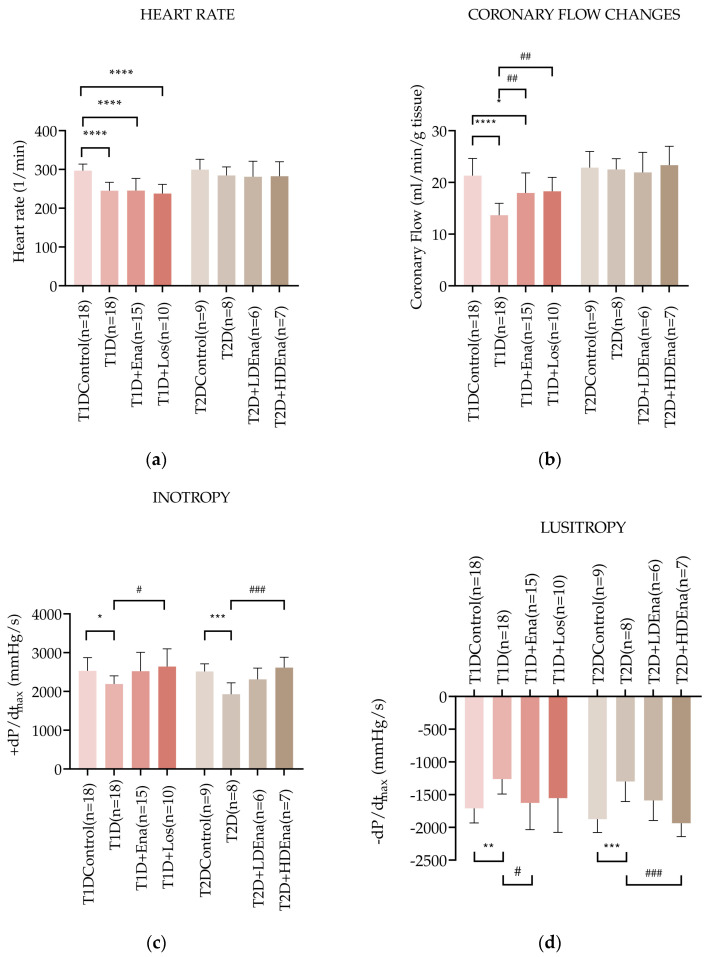
Hemodynamic parameters of isolated hearts in T1D and T2D models. Data of heart rate (**a**), coronary flow (**b**), left ventricular inotropy: +dP/dt_max_ (**c**), and left ventricular lusitropy: −dP/dt_max_ (**d**) in T1D Control (*n* = 18), T1D (*n* = 18), T1D + Ena (*n* = 15) and T1D + Los (*n* = 10), T2D Control (*n* = 9), T2D (*n* = 8), T2D + LD Ena (*n* = 6) and T2D + HD Ena (*n* = 7) groups. Statistics: one-way ANOVA and Tukey’s multiple comparison post hoc test; *: *p* < 0.05, **: *p* < 0.01, ***: *p* < 0.001, ****: *p* < 0.0001 vs. T1D Control or T2D Control values, ^#^: *p* < 0.05, ^##^: *p* < 0.01, ^###^: *p* < 0.001 vs. values in diabetic (T1D or T2D) groups. (Mean ± SD). Abbreviations: T1D—type 1 diabetes, T2D—type 2 diabetes, +dP/dt_max_—left ventricular inotropy, −dP/dt_max_—left ventricular lusitropy, Ena—Enalapril, Los—Losartan, LD Ena—low-dose Enalapril, HD Ena—high-dose Enalapril.

Figure 3a shows heart rate data in T1D and T2D models. The heart rate in the T1D group was significantly reduced compared to Control (245 ± 22 vs. 297 ± 17 beats/min alternatively, respectively; *p* < 0.0001). Neither Enalapril nor Losartan treatment prevented the decrease in heart rate in T1D animals. Heart rate data did not change significantly in any of the experimental groups of T2D.

Changes in the coronary flow of isolated rat hearts are illustrated in Figure 3b. Coronary flow was reduced in T1D hearts (T1D: 13.7 ± 2.3 vs. Control: 21.3 ± 3.3 mL/min; *p* < 0.0001). This reduction was significantly mitigated by both Enalapril and Losartan treatments. Coronary flow data did not differ significantly from each other in any of the T2D groups (Figure 3b).

Figure 3c presents the +dP/dt_max_ values indicating left ventricular inotropy in T1D and T2D hearts. Cardiac contractility was reduced in T1D isolated hearts compared to Control (+dP/dt_max_: 2192 ± 208 vs. 2532 ± 341 mmHg/s, respectively; *p* < 0.05). In both T1D + Ena and T1D + Los groups, Enalapril and Losartan treatments prevented the impairment of contractility since there was no significant difference between the Control and the treated groups. Contractility was significantly elevated in the T1D + Los group compared to the T1D state and showed considerable improvement in the T1D + Ena group (*p* = 0.063). In T2D, +dP/dt_max_ was also reduced compared to Control (T2D: 1930 ± 291 vs. Control 2514 ± 197 mmHg/s; *p* < 0.001). Altogether, the decreased inotropy in T2D was somewhat mitigated by LD Enalapril (borderline significance in the T2D + LD Ena group compared to T2D (*p* = 0.0524) and was prevented by HD Enalapril treatments (no statistically significant differences between Control and T2D + LD Ena and T2D + HD Ena groups, Figure 3c).

Figure 3d displays the −dP/dt_max_ values, which indicate left ventricular lusitropic function in T1D and T2D isolated hearts. In T1D, the relaxation ability was impaired compared to Control (−dP/dt_max_: −1261 ± 230 vs. −1710 ± 223 mmHg/s, respectively; *p* < 0.01), which was corrected by both Enalapril and Losartan treatments. Although the elevation of −dP/dt_max_ reached a statistically significant difference only in the T1D + Ena group compared to T1D, there were no significant differences between the Control and T1D + Ena and T1D + Los groups. In T2D, −dP/dt_max_ also decreased significantly compared to Control (T2D: −1299 ± 305 vs. Control: −1874 ± 205 mmHg/s; *p* < 0.001). Both LD and HD Enalapril treatments improved relaxation capacity (Figure 3d). Although the improvement of lusitropy in response to treatments was statistically significant only in the T2D + HD Ena group compared to T2D, the partial restoration of the −dP/dt_max_ parameter was also evident in the T2D + LD Ena group because there was no statistical difference between this and the Control group.

### 3.5. Myocardial Ca^2+^_i_ Handling in Langendorff Hearts of T1D and T2D Animals

**Figure 4:**
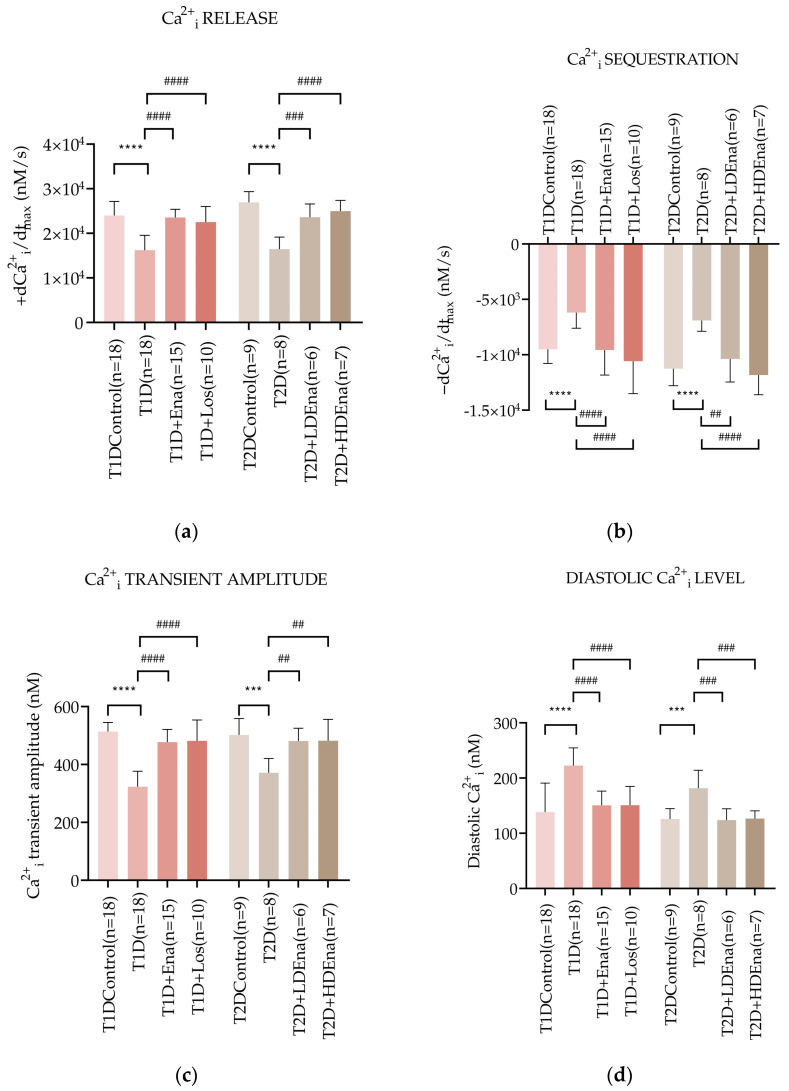
Changes in myocardial Ca^2+^_i_ transients in T1D and T2D animals. Alterations of Ca^2+^_i_ release: +dCa^2+^_i_/dt_max_ (**a**), Ca^2+^_i_ sequestration: -dCa^2+^_i_/dt_max_ (**b**), Ca^2+^_i_ transient amplitude (**c**) and diastolic Ca^2+^_i_ level (**d**) in T1D Control (*n* = 18), T1D (*n* = 18), T1D + Ena (*n* = 15) and T1D + Los (*n* = 10), T2D Control (*n* = 9), T2D (*n* = 8), T2D + LD Ena (*n* = 6) and T2D + HD Ena (*n* = 7). Statistics: one-way ANOVA and Tukey’s multiple comparison post hoc test; ***: *p* < 0.001, ****: *p* < 0.0001 vs. Control values, ^##^: *p* < 0.01, ^###^: *p* < 0.001, ^####^: *p* < 0.0001 vs. values in diabetic (T1D or T2D) groups. (Mean ± SD). Abbreviations: Ca^2+^_i_—intracellular calcium, T1D—type 1 diabetes, T2D—type 2 diabetes, +dCa^2+^_i_/dt_max_—Ca^2+^_i_ release, -dCa^2+^_i_/dt_max_—Ca^2+^_i_ sequestration, Ena—Enalapril, Los—Losartan, LD Ena—low-dose Enalapril, HD Ena—high-dose Enalapril.

Figure 4a presents +dCa^2+^_i_/dt_max_ data characterizing the dynamics of Ca^2+^_i_ release from the SR of the cardiomyocytes in Langendorff-perfused hearts. In T1D, +dCa^2+^_i_/dt_max_ decreased by almost 32% compared to Control (16,230 ± 3324 vs. 23,972 ± 3168, respectively mM/s; *p* < 0.0001). This decrease was prevented by the ACE inhibitor Enalapril (T1D + Ena group) and the AT1R blocker Losartan (T1D + Los group). In T2D, Ca^2+^_i_ release from the SR was also reduced compared to Control (T2D: 16,465 ± 2692 vs. Control: 26,937 ± 2397 vs. mM/s; *p* < 0.0001). Both LD and HD Enalapril treatments normalized Ca^2+^_i_ release from the SR (T2D + LD Ena and T2D + HD Ena groups, Figure 4a).

Figure 4b displays −dCa^2+^_i_/dt_max_ data, which represents the dynamics of Ca^2+^_i_ sequestration by SERCA2a back to the SR of the cardiomyocytes in isolated, perfused hearts. In T1D, the maximum rate of Ca^2+^_i_ removal slowed down by almost 35% compared to Control (−6193 ± 1415 vs. −9511 ± 1270 mM/s, respectively; *p* < 0.0001). However, Enalapril and Losartan treatments normalized these changes (T1D + Ena and T1D + Los groups). In T2D, the Ca^2+^_i_ sequestration also deteriorated (T2D: −6905 ± 975 vs. Control: −11,259 ± 1534 mM/s; *p* < 0.0001), however both LD and HD Enalapril treatments could restore the dynamics of Ca^2+^_i_ removal in T2D (T2D + LD Ena and T2D + HD Ena groups, Figure 4b).

The pathological disturbances in Ca^2+^_i_ release and sequestration affected the peak Ca^2+^_i_ rise during systole and the resting Ca^2+^_i_ level during diastole in cardiomyocytes. Figure 4c shows data on the Ca^2+^_i_ transient amplitude in the cardiomyocytes of isolated hearts. T1D produced a marked decline of the Ca^2+^_i_ transient amplitude compared to Control (323.27 ± 53.50 vs. 513.61 ± 31.89 mM, respectively; *p* < 0.0001). Both Enalapril and Losartan treatments preserved the Ca^2+^_i_ transient amplitude in T1D + Ena and T1D + Los groups. A reduced Ca^2+^_i_ transient amplitude was also observed in T2D hearts compared to Control (T2D: 371.04 ± 49.81 vs. 502.14 ± 56.41 mM; *p* < 0.001). This reduction was prevented by both LD and HD Enalapril treatments (T2D + LD Ena and T2D + HD Ena groups, Figure 4c).

Changes in diastolic Ca^2+^_i_ levels in cardiomyocytes of perfused hearts are displayed in Figure 4d. The diastolic Ca^2+^_i_ level was considerably elevated in T1D hearts compared to Control (222.66 ± 31.94 vs. 138.26 ± 52.68 mM, respectively; *p* < 0.0001). Enalapril or Losartan treatment successfully prevented this increase (T1D + Ena and T1D + Los groups). In T2D, the diastolic Ca^2+^_i_ level also increased significantly compared to Control (T2D: 181.61 ± 32.61 vs. Control: 125.94 ± 18.75 mM; *p* < 0.001), which elevation was reduced back to Control by both LD and HD Enalapril treatments (T2D + LD Ena and T2D + HD Ena groups, Figure 4d).

### 3.6. Expression of Key Ca^2+^_i_ Cycling Enzymes in T2D Animals

**Figure 5:**
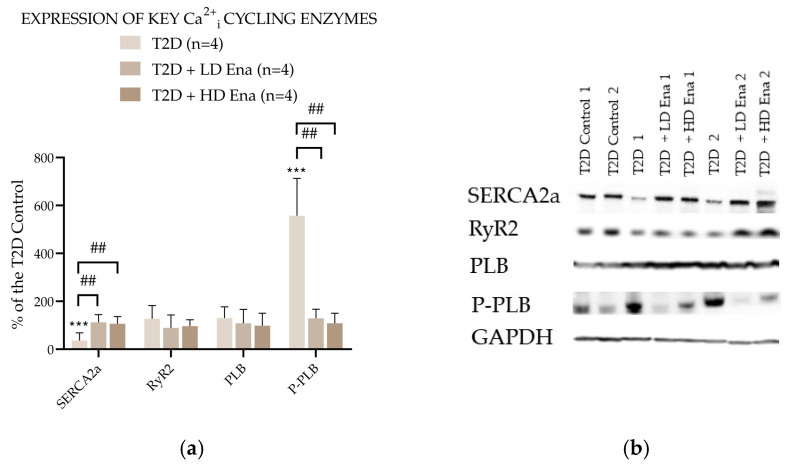
Expression of key Ca^2+^_i_ cycling enzymes in T2D animals: Protein abundance of RyR2, SERCA2a, PLB, and P-PLB normalized to GAPDH and expressed as a percentage of the values measured in T2D Control (*n* = 4 in each group) (**a**). Representative Western blot images (**b**). Statistics: Kruskal–Wallis ANOVA on ranks and Dunn’s multiple comparison test. ***: *p* < 0.001 vs. T2D Control values; ^##^: *p* < 0.01 vs. T2D; (Mean ± SD). Abbreviations: Ca^2+^_i_—intracellular calcium, T2D—type 2 diabetes, RyR2—Ryanodine receptor type 2, SERCA2a—Sarcoendoplasmic reticulum Ca^2+^-ATPase 2a, PLB—Phopholamban, P-PLB—16Ser-phosphorylated phospholamban, GAPDH—Glyceraldehyde 3-phosphate dehydrogenase.

Figure 5 presents the abundance of Ca^2+^_i_ cycling proteins expressed as a percentage of the Control value in the T2D experimental groups. The expression level of RyR2 in T2D did not differ from that measured in T2D Control and was not affected by either LD or HD Enalapril treatments. However, SERCA2a abundance was significantly lower in the T2D group compared to the T2D Control and normalized in the Enalapril-treated groups. The level of SERCA2a regulator PLB was unaffected by T2D and remained unchanged in the Ena-treated groups. However, its phosphorylated form—P-PLB—was detectable in significantly higher amounts in the heart samples of the T2D group. This rise in P-PLB abundance was not observable in the T2D + LD Ena and T2D + HD Ena groups.

## 4. Discussion

The main finding of this study is that the development of diabetic cardiomyopathy interferes with normal Ca^2+^_i_ handling of the cardiomyocytes of isolated, perfused rat hearts, and the development of these Ca^2+^_i_ homeostasis disturbances can be prevented by blocking the activity of the RAS system in either T1D or T2D pathological states. Consequently, the restoration of normal Ca^2+^_i_ cycling in the cardiomyocytes by suppression of local RAS activity drives the recuperation of the hemodynamic performance of the diabetic heart. Chronic suppression of ATII activity by ACE inhibition or AT1R blockade mitigates heart failure symptoms, most probably by the suppression of local RAS in the cardiac tissue. Our study is, to our knowledge, the first to provide evidence in an intact heart model that disrupted cardiac Ca^2+^_i_ handling may be a key factor in RAS-mediated alterations in diabetic heart failure.

### 4.1. Isolated Pefused Heart Model

The development of diabetic cardiomyopathy seriously hampers the life expectancy of T1D and T2D patients. Although diabetic heart failure is the result of a multifactorial pathological chain of events, abnormal Ca^2+^_i_ homeostasis in the cardiomyocytes is a plausible explanation for the worsening mechanical events developing in the heart during this disease. Disturbances of inotropic and lusitropic functions of the heart are related to altered characteristics of the Ca^2+^_i_ transient. In the past, several studies aimed to describe the relationship between changes in myocardial Ca^2+^_i_ handling and contractile dysfunction [46,47,48]. However, most of these experiments were carried out in isolated cardiomyocytes, which lack their natural environment compared to myocytes functioning in a whole organ with intact circulation [49]. In our work, an ex vivo intact perfused heart preparation was combined with surface fluorometry to detect changes in Ca^2+^_i_ homeostasis together with hemodynamic parameters, whose experimental condition is closer to the in vivo situation than isolated myocardial cell models [50]. The simultaneous detection of Ca^2+^_i_ transient and inotropic and lusitropic functional parameters in intact myocardial tissue provides an excellent tool to analyze the effects of diabetes-associated pathological events within the cardiomyocytes on complex heart function. Specifically, the consequences of local RAS activation induced by T1D or T2D on the contractile performance of the heart muscle in direct connection with Ca^2+^_i_ handling can be reliably investigated in our isolated perfused heart model.

### 4.2. Validation of T1D and T2D Models

Our T1D rat model displays the consequences of insulin deficiency induced by streptozotocin treatment, i.e., decreased body weight and heart weight and chronic severe hyperglycemia [51,52,53] (Table 1). Local activation of the RAS in the different tissues undoubtedly contributes to growth restriction and tissue atrophy by inducing apoptosis and fibrosis since body weight and heart weight were significantly higher after either Enalapril or Losartan treatment. On the other hand, blood glucose levels were hardly affected by these drugs, indicating that the hyperglycemia in streptozotocin-induced T1D is mainly due to the lack of primary action of insulin on peripheral glucose uptake [15].

Our fructose-fed T2D model displays the classic symptoms of metabolic syndrome related to insulin-resistant diabetes, i.e., higher than normal mean arterial pressure, increased heart weight, and a heart-weight-to-body-weight ratio indicating myocardial hypertrophy (Table 1). Although fasting blood glucose levels in our T2D experimental groups did not differ from Controls, insulin resistance was indicated by significantly elevated fasting insulin levels and increased 2 h blood glucose and insulin levels after the oGTT. These changes confirm the T2D condition, consistent with findings in fructose-fed diabetic animals reported by others (Figure 2) [44,45]. The increase in heart weight was also confirmed by echocardiography, demonstrating significantly higher diastolic wall thickness values (Table 2). This myocardial hypertrophy can be primarily attributed to chronically elevated mean arterial pressure evoked by ATII acting via the AT1 receptor. The hypertrophy is potentiated by cardiac RAS activation and the consequent induction of cell proliferation, inflammatory responses, and pro-fibrotic processes, collectively contributing to myocardial remodeling [21,54]. Diet-induced metabolic changes were not affected by the inhibition of RAS, but our model responded to Ena treatment as expected, showing a reduction in myocardial hypertrophy and blood pressure in response to chronic HD (depressor) Ena administration.

Echocardiography data confirm the development of diabetic cardiomyopathy both in T1D and T2D since the dynamic pump function values of ejection fraction and fractional shortening were significantly depressed compared to Controls (Table 2). The depression of EF and FS parameters underlying the state of diabetic cardiomyopathy was also evidenced by others [55,56]. Our data also support those of others showing depressed contractile activity in isolated cardiomyocytes [57] or in intact hearts [25]. These data also suggest that local RAS activation in the myocardial tissue interferes with normal contractile activity because cardiac performance was improved by the inhibition of RAS in response to either Enalapril or Losartan treatment in T1D and was rescued by HD Enalapril treatment in T2D.

### 4.3. Local RAS Activation in Diabetes

Diabetic cardiomyopathy is associated with the local activation of RAS in the cardiac tissue, which leads to enhanced fibrosis and tissue remodeling [58]. In this study, we report findings obtained from an isolated heart preparation with intact circulation, strongly supporting that local RAS activation in cardiac tissue under diabetic conditions (T1D or T2D) impairs the heart’s inotropic and lusitropic functions. Concurrently, the parameters of Ca^2+^_i_ release and sequestration by the SR also deteriorate. However, these disturbances of the myocardial Ca^2+^_i_ transient in diabetes were effectively counteracted by the ACE inhibitor Enalapril or the AT1R receptor antagonist Losartan treatment. In T1D, chronic Enalapril or Losartan treatment prevented the reduction in the rate of rise and rate of decline of the Ca^2+^_i_ transient, and pump function was maintained at a normal level. In T2D, disturbances of the Ca^2+^_i_ transient and the contractile parameters were effectively mitigated by LD Enalapril and were completely normalized by HD Enalapril treatment. The observation that even subdepressor (LD) Enalapril treatment was beneficial in this regard, which failed to normalize blood pressure and thereby exclude the direct effects of increased afterload and systemic RAS on cardiac remodeling, strongly suggests that the beneficial effects of RAS blockade observed in our study are primarily attributable to the suppression of local tissue RAS pathways. The likely role of local RAS is also supported by studies that reported normal or depressed plasma renin activity along with overactivation of RAS at tissue level [59,60,61]. Our observation could be further supported by measurements of plasma renin activity and determination of ATII levels, renin activity, and expression level of RAS components in the cardiac tissue [62,63] in our model; however, this was not part of our study. Our observations in perfused hearts under T1D conditions are in line with those of Turan et al. [57] obtained in isolated STZ-induced diabetic cardiomyocytes, which showed normalization of the altered Ca^2+^_i_ transient and contractile activity in response to AT1R antagonist treatment.

During the activation of local RAS, ATII production has been shown to occur both intra- and extracellularly [58,64]. As indicated by the in vitro studies of Kumar et al., cardiomyocytes predominantly secrete intracellular ATII via renin and chymase pathways, whereas ATII production by cardiac fibroblasts takes place both intra- and extracellularly via renin and angiotensin-converting enzyme pathways [20]. The equally effective ACE inhibitor and AT1R blocker treatment in our T1D model indicate that extracellular ACE has an important role in the activation of local, cardiac RAS and the production of ATII in STZ-induced diabetes since the applied ACE inhibitor cannot enter cells and affect intracellular ATII production in the cardiomyocytes. A similar conclusion can be drawn from our results obtained in T2D in response to HD Enalapril treatment preventing extracellular ATII production. Altogether, these results indicate that extracellular ACE activation and ATII production, most probably by cardiac fibroblasts, play an important role in diabetes-induced alterations of Ca^2+^_i_ homeostasis and, consequently, contractile performance of the isolated rat heart.

### 4.4. Hemodynamic Parameters in Langendorff Hearts

The heart rate in Langendorff-perfused hearts changed differently in T1D and T2D (Figure 3a). In T1D hearts, heart rate was significantly lower than in Controls, regardless of Enalapril or Losartan treatment. This finding corroborates earlier studies showing lower heart rates in isolated rat hearts of experimental diabetes as a sign of negative chronotropy in insulin deficiency [53,65]. Our results also show that this negative chronotropic effect of T1D is independent of enhanced ATII activity in the cardiac tissue. Although insulin resistance was evident in our T2D model (Figure 2), the reduced insulin effect was not associated with decreased heart rate, in contrast to the insulin-deficient T1D model (Figure 3a). This observation is again in line with other studies in T2D models [29,66] and also shows that chronic RAS inhibition by ACE does not affect the heart rate status of Langendorff hearts in our T2D model. These findings suggest that pacemaker activity of the diabetic heart is independent of local RAS activity.

Both inotropy and lusitropy were significantly impaired in T1D hearts but did not differ significantly from T1D Control in response to Enalapril and Losartan treatments (Figure 3c,d). A possible explanation is that hyperglycemia-induced local ATII increases oxidative stress through a positive feedback mechanism, further enhancing the expression of RAS components, including extracellular ATII [58]. Extracellular ATII causes enhancement of oxidative stress and cardiac fibrosis via AT1Rs [64,67,68] that may interfere with normal Ca^2+^_i_ cycling (see Section 4.5), which altogether impairs the normal contractile activity of the cardiomyocytes (Figure 3c,d). The development of reduced inotropy and lusitropy could be prevented by chronic supplementation with an ACE inhibitor or AT1 receptor blocker. Enalapril and Losartan presumably exert this favorable effect by inhibiting extracellular ATII generation by fibroblasts [58]. In our study, the contribution of fibrosis to contractile dysfunction has not been assessed. However, several studies have proven the detrimental effects of fibrosis on cardiac function and the beneficial anti-fibrotic effects of ACE inhibitors in similar experimental models and in human studies [68,69,70,71,72,73,74].

Data of coronary flow seemed to follow changes in contractile activity, hence the actual O_2_ need of the myocardial tissue in the T1D experimental groups (Figure 3b). The decline in +dP/dt_max_ and −dP/dt_max_ observed after STZ treatment, along with their restoration in response to Enalapril and Losartan treatments, was accompanied by a reduction in CF followed by a trend toward recovery to Control values. This reduction in O_2_ need, hence CF, seems to be amplified by the negative chronotropy developing in insulin deficiency, at least in the basal state. Conversely, the reduced CF in T1D animals may also stem from the characteristic endothelial dysfunction associated with diabetes, which, in turn, contributes to impaired contractile performance [75]. This vascular dysfunction may be partly linked to RAS activation-induced oxidative stress [76] and can, therefore, be alleviated by RAS blockade treatments.

The deterioration of pump activity was also evident in our T2D-perfused heart model (Figure 3c,d). The negative inotropy and lusitropy observed in this state were most probably related to the activation of local RAS since LD Ena treatment partially restored, whereas HD Ena treatment completely prevented the reduction in contractile performance. Contractility in T2D could be impaired by the effects of cardiac RAS inducing apoptosis, fibrosis, and remodeling on the one hand [64] and by affecting normal Ca^2+^_i_ handling within the cardiomyocytes on the other (see Section 4.5). Interestingly, coronary flow was not reduced in the basal T2D state compared to Control (Figure 3b) and remained unaffected by LD or HD Ena treatment. These data may indicate that the lack of negative chronotropy in this model weighs heavily in determining the O_2_ need of the myocardium, hence CF, at least in the basal state.

### 4.5. Myocardial Ca^2+^_i_ Transients in Langendorff Hearts

Pathological alterations of the parameters of the Ca^2+^_i_ transients correlated well with reduced left ventricular inotropic and left ventricular lusitropic functions (Figure 4). Data of +dCa^2+^_i_/dt_max_ and −dCa^2+^_i_/dt_max_ are indices of Ca^2+^_i_ release from the SR via the ryanodine channel and sequestration back to the SR by the SERCA2a, respectively. Both in T1D and T2D hearts, Ca^2+^_i_ release and sequestration were significantly slower in the basal pathological state underlying reduced inotropy and lusitropy (Figure 3c,d) and representing decreased RyR2 and SERCA2a activity, respectively. Furthermore, decreased activity of the RyR2 channels leads to reduced amplitude of the Ca^2+^_i_ transient, whereas slower SERCA2a pump activity results in elevated diastolic Ca^2+^_i_ levels (Figure 4c,d). Our findings are consistent with previous studies conducted in TD1 models. Ligeti et al. reported the impairment of SERCA2a function in STZ-induced diabetes, as indicated by a decrease in the transporter V_max_ in the early phase of the disease [25]. Decreased levels of RyR2 and SERCA proteins in T1D diabetic cardiomyocytes have also been reported by others [24].

The protein expression levels of Ca^2+^_i_ regulating enzymes showed no changes in RyR2 in the T2D models, indicating that alterations in Ca^2+^_i_ release dynamics in our model are most probably contributable to defects in RyR2 function rather than changes in its expression level. This is in line with studies that suggested RYR2 dysfunction without relevant changes in its expression in diabetic models [77,78]. However, T2D was associated with decreased SERCA2a expression, consistent with several previous studies that also reported a significant reduction in SERCA2a levels in both T1D and T2D [24,55,79,80,81,82]. Expression levels of P-PLB may indicate that compensatory mechanisms counter-regulate the loss of SERCA2a pump activity, as enhanced phosphorylation of PLB releases SERCA2a from PLB inhibition. However, this adaptation failed to restore the dynamics of Ca^2+^_i_ removal. These changes in P-PLB expression are consistent with our previous findings obtained in the same model at an early stage of the disease [29]. Interestingly, these changes were completely prevented when Enalapril treatment (either at LD or HD) was applied. A possible explanation could be that T2D-related overactivity of brain RAS enhances sympathetic outflow to the cardiovascular system, thereby not only contributing to the development and maintenance of hypertension but also promoting excessive phosphorylation of PLB [83]. This ATII-mediated effect is likely to be attenuated by Ena in our study, mitigating the downstream physiological consequences.

The cardiac remodeling induced by local RAS activation may well be associated with depressed SR function in T1D and T2D cardiomyocytes. The enhanced local production of ATII can interfere with normal RyR2 and SERCA2a functions by activating signal transduction pathways, which may lead to suppression of RyR2 function and reduced SERCA2a expression and/or activity in the diabetic cardiomyocytes. Hence, interruption of the ACE-ATII pathway may ameliorate the contractile performance of the beating heart by alleviating disturbances of Ca^2+^_i_ handling in diabetic cardiomyocytes. In our study, impairment of RyR2 and SERCA2a functions were normalized by ACE inhibition and AT1R antagonist treatments (Figure 4a,b), and concomitantly normal contractile activities were restored. Also, administration of Enalapril or Losartan in the diet of the experimental animals restored the amplitude of the Ca^2+^_i_ transient and the normal diastolic Ca^2+^_i_ level (Figure 4c,d). These findings indicate that elevated ATII levels in cardiac tissue disrupt normal RyR2 and SERCA2a activities, linking enhanced myocardial RAS activity in T1D and T2D to disturbances in cardiac Ca^2+^_i_ homeostasis.

The mechanistic link between RAS activation and impaired Ca^2+^_i_ cycling may occur both at a functional and an expressional level. The study by Turan B. et al. suggests oxidative stress as a potential mediator of the relationship, as they found that antioxidants reduced Ca^2+^_i_ concentrations in cardiac myocytes of STZ-induced diabetic rats, with similar results observed in isolated papillary muscles from diabetic animals treated with AT1R blockers, leading to normalized cardiac Ca^2+^_i_ transients and improved cardiomyocyte contractile activity [57]. Indeed, RAS activation and ATII enhance oxidative stress via several pathways, particularly by activating NADPH oxidase 4 (NOX4), leading to excessive production of reactive oxidative species, disruption of mitochondrial function, stimulation of proinflammatory Nuclear Factor kappa B (NF-κB) pathways, and suppression of Nuclear Factor Erythroid 2-Related Factor 2 (Nrf2) related antioxidant defense [21,84]. Oxidative stress has been shown to interfere with the function of RyR2, and SERCA2a by oxidative posttranslational modifications (carbonylation, disulfide bond formation, hydroxylation) of the channels/transporters [78,85,86,87]. In addition, hyperglycemia-induced formation of advanced glycation end products on RYR2 and SERCa2a may also contribute [88,89]. Moreover, AT1R activation by ATII stimulates downstream pathways that activate NF-κB [21]. Nuclear translocation of NF-κB has been linked to reduced SERCA2a gene promoter activity, resulting in decreased expression of the transporter and diastolic dysfunction [90].

### 4.6. Therapeutic Implications

The use of ACE inhibitors and ATR1 blockers has been extensively studied in diabetic patients with heart failure. The LIFE study demonstrated that losartan reduced cardiovascular morbidity and mortality in diabetic patients compared to atenolol, showing that it has benefits beyond blood pressure reduction [22]. Similarly, the HOPE and MICRO-HOPE trials highlighted the role of ACE inhibitors in reducing cardiovascular events and microvascular complications. Among diabetic patients, ramipril use was associated with a significant 25% reduction in risk for the composite endpoint of myocardial infarction, stroke, or cardiovascular death after a median follow-up period of 4.5 years, and this benefit was independent of any blood pressure-lowering effect [23,91]. However, some large studies and meta-analyses have not consistently confirmed additional cardiovascular benefits or decreased mortality in diabetic patients treated with these agents [92,93,94,95,96,97,98,99]. Our study provides further insight into this ongoing debate by emphasizing the potential advantages of ATR1 blockers and ACE inhibitors in managing heart failure in diabetes, and our findings reinforce the importance of optimizing RAS-targeted therapies to improve cardiac outcomes in diabetic patients.

### 4.7. Limitations of the Study

A limitation of our study is that it involved only male rats. However, considering the differences in the mechanisms of heart failure between females and males [100,101], the observations must be confirmed in female individuals, too. In addition, our study delineates the relationship between RAS activation and disrupted Ca^2+^_i_ cycling in the diabetic heart; however, further research is needed to provide evidence concerning the connecting cellular mechanism and the concurrent morphological status of the myocardium.

## 5. Conclusions and Future Perspectives

The findings of this study suggest that RAS activation, most likely occurring at the tissue level, plays a key role in disrupting myocardial Ca^2+^_i_ regulatory mechanisms and contributes to the development of diabetic cardiomyopathy in T1D and T2D. These observations also underline the importance of suppression of RAS activity in the cardiac tissue by ACE inhibitor or AT1R antagonist treatment. The effects of ATII on cardiac Ca^2+^_i_ homeostasis are mainly mediated through AT1R signaling pathways, but the details of the signaling cascade in diabetic cardiomyopathy need further clarification. Considering the increasing prevalence of diabetes and the lack of specific therapies for diabetic cardiomyopathy, further research is warranted to establish the role of available heart failure treatments in diabetic patients. In this context, our results contribute to a better understanding of the role of RAS inhibitor therapies.

## Figures and Tables

**Table 1:** General physiological parameters and glucose levels in T1D and T2D groups. This table summarizes the physiological parameters such as body weight, heart weight, heart weight to body weight ratio, mean arterial pressure and blood glucose levels of animals in T1D Control (*n* = 18), T1D (*n* = 18), T1D + Ena (*n* = 15) and T1D + Los (*n* = 10), T2D Control (*n* = 9), T2D (*n* = 8), T2D + LD Ena (*n* = 6) and T2D + HD Ena (*n* = 7) groups. Statistics: one-way ANOVA and Tukey’s multiple comparison post hoc test; * *p* < 0.05; ** *p* < 0.01; *** *p* < 0.001; **** *p* < 0.0001 vs. T1D Control or T2D Control values; ^##^: *p* < 0.01; ^###^ *p* < 0.001; ^####^ *p* < 0.0001 vs. values in diabetic (T1D or T2D) groups; ^$^ *p* < 0.05; ^$$^ *p* < 0.01; vs. values in T2D + LD Ena group (Mean ± SD). Abbreviations: T1D—type 1 diabetes, T2D—type 2 diabetes, Ena—Enalapril, Los—Losartan, LD Ena—low-dose Enalapril, HD Ena—high-dose Enalapril.

	T1D Control (*n* = 18)	T1D (*n* = 18)	T1D + Ena (*n* = 15)	T1D + Los (*n* = 10)
Body weight (g)	403 ± 18	194 ± 24 ****	274 ± 53 ****^,####^	287 ± 66 ****^,####^
Heart weight (g)	1.6 ± 0.2	0.8 ± 0.1 ****	1.2 ± 0.2 ****^,####^	1.2 ± 0.3 ****^,###^
Heart weight/Body weight (×10^−3^)	4.1 ± 0.6	4.2 ± 0.9	4.4 ± 1.1	4.4 ± 1.0
Mean arterial pressure (mmHg)	87 ± 9	82 ± 7	80 ± 11	85 ± 9
Blood glucose (mmol/L)	8.6 ± 2.2	22.2 ± 3.4 ****	21.4 ± 4.1 ****	23.6 ± 6.8 ****
	**T2D Control** **(*n* = 9)**	**T2D** **(*n* = 8)**	**T2D + LD Ena** **(*n* = 6)**	**T2D + HD Ena** **(*n* = 7)**
Body weight (g)	511 ± 27	507 ± 35	508 ± 33	493 ± 29
Heart weight (g)	1.8 ± 0.2	2.2 ± 0.2 **	2.1 ± 0.3 *	1.7 ± 0.2 ^##,$^
Heart weight/Body weight (×10^−3^)	3.5 ± 0.4	4.4 ± 0.2 ***	4.1 ± 0.4 *	3.4 ± 0.3 ^###,$$^
Mean arterial pressure (mmHg)	88 ± 10	122 ± 7 ****	117 ± 15 ****	89 ± 9 ^###,$$^
Fasting blood glucose (mmol/L)	7.3 ± 4.1	6.7 ± 3.4	7.7 ± 3.8	8.1 ± 4.2

**Table 2:** Echocardiography data in T1D and T2D. This table summarizes the echocardiographic data of diastolic wall thickness, end-diastolic volume, ejection fraction and fractional shortening of hearts in T1D Control (*n* = 18), T1D (*n* = 18), T1D + Ena (*n* = 15) and T1D + Los (*n* = 10), T2D Control (*n* = 9), T2D (*n* = 8), T2D + LD Ena (*n* = 6) and T2D + HD Ena (*n* = 7) groups. Statistics: one-way ANOVA and Tukey’s multiple comparison post hoc test; * *p* < 0.05; ** *p* < 0.01; *** *p* < 0.001 vs. T1D Control or T2D Control values; ^#^ *p* < 0.05; vs. values in diabetic (T1D or T2D) groups; ^$^ *p* < 0.05 vs. values in T2D + LD Ena group (Mean ± SD). Abbreviations: T1D—type 1 diabetes, T2D—type 2 diabetes, Ena—Enalapril, Los—Losartan, LD Ena—low-dose Enalapril, HD Ena—high-dose Enalapril, IVSd—Diastolic wall thickness, EDV—End-diastolic volume, EF—Ejection fraction, FS—Fractional shortening, LVIDd—left ventricular internal diameters in diastole, LVIDs—left ventricular internal diameters in systole.

	T1D Control (*n* = 18)	T1D (*n* = 18)	T1D + Ena (*n* = 15)	T1D + Los (*n* = 10)
Diastolic wall thickness (IVSd) (cm)	0.18 ± 0.04	0.17 ± 0.04	0.19 ± 0.03	0.18 ± 0.03
End-diastolic volume (EDV) (cm^3^)	0.34 ± 0.07	0.30 ± 0.06	0.33 ± 0.05	0.32 ± 0.07
Ejection fraction (EF: 100 × SV/EDV) (%)	79 ± 7	54 ± 11 ***	65 ± 10 *^,#^	69 ± 10 *^,#^
Fractional shortening (FS: 100 × (LVIDd-LVIDs)/LVIDd) (%)	50 ± 6	40 ± 5 **	45 ± 9	47 ± 6 ^#^
	**T2D Control** **(*n* = 9)**	**T2D** **(*n* = 8)**	**T2D + LD Ena** **(*n* = 6)**	**T2D + HD Ena** **(*n* = 7)**
Diastolic wall thickness (IVSd) (cm)	0.19 ± 0.04	0.26 ± 0.03 **	0.25 ± 0.04 **	0.21 ± 0.03 ^#,$^
End-diastolic volume (EDV) (cm^3^)	0.40 ± 0.07	0.43 ± 0.10	0.42 ± 0.06	0.44 ± 0.08
Ejection fraction (EF: 100 × SV/EDV) (%)	73 ± 8	52 ± 6 **	62 ± 8 *^,#^	76 ± 8 ^#,$^
Fractional shortening (FS: 100 × (LVIDd-LVIDs)/LVIDd)(%)	48 ± 6	38 ± 7 ***	41 ± 7 *^,#^	47 ± 6 ^#,$^

## Data Availability

The raw data supporting the conclusions of this article will be made available by the authors upon request.

## Abbreviations

+dCa^2+^_i_/dt_max_	Ca^2+^_i_ release
+dP/dt_max_	Left ventricular inotropy
ACE	Angiotensin-converting enzyme
AT1R	Angiotensin type 1 receptor
ATII	Angiotensin II
Ca^2+^_i_	Intracellular calcium/intracellular calcium
−dCa^2+^_i_/dt_max_	Ca^2+^_i_ sequestration
−dP/dt_max_	Left ventricular lusitropy
EDV	End-diastolic volume
EF	Ejection fraction
Ena	Enalapril
ESV	End-systolic volume
FS	Fractional shortening
HD Ena	High-dose enalapril
IVSd	Diastolic wall thickness
LD Ena	Low-dose enalapril
Los	Losartan
LVA	Left ventricular area
LVIDd	Left ventricular internal diameters in diastole
LVIDs	Left ventricular internal diameters in systole
LVL	Left ventricular length
MAPKs	Mitogen-activated protein kinase
NF-κB	Nuclear factor-kappa B
NOX	NADPH oxidase
oGTT	Oral glucose tolerance test
PLB	Phospholamban
PLC	Phospholipase C
P-PLB	16Ser-phosphorylated phospholamban
RAS	Renin–angiotensin system
RyR2	Ryanodine receptor type 2
SERCA2a	Sarcoendoplasmic reticulum Ca^2+^-ATPase 2a
SR	Sarcoplasmic reticulum
STZ	Streptozotocin injection
T1D	Type 1 diabetes
T2D	Type 2 diabetes
